# Effects of Relationship Type on Quality of Life in Older Adults with Cognitive Impairment and the Informal Caregivers

**DOI:** 10.1093/geroni/igab046.3593

**Published:** 2021-12-17

**Authors:** Aiping Lai, Julie Richardson, Lauren Griffith, Ayse Kuspinar, Jenna Smith-Turchyn

**Affiliations:** 1 McMaster University, Hamilton, Ontario, Canada; 2 McMaster University, McMaster University, Ontario, Canada

## Abstract

Purpose: The purpose of this study was to assess whether there was an association between care-recipient relationship type and the QoL of older adults and their informal caregivers, and whether this association pertained to older adults’ cognitive function. Methods: This was a secondary data analysis. Older adults (n=1230) and their informal caregivers (n=1871) were identified from participants in the National Health and Aging Trends Study (NHATS) Round 5 and the National Study of Caregiving (NSOC) II. A series of bivariate and multivariable regression models examined the associations among the care-recipient relationship type and QoL in older adults and their informal caregivers, adjusted for socio-demographic variables as well as cognitive functioning. Results: Both older adults and caregivers’ QoL outcomes varied by the type of relationship. Recipients cared for by adult-child caregivers or multiple caregivers experienced higher functional limitation than those cared by spousal caregivers (β=.79, CI [.39, 1.19]; β=.50, CI [.17, .82], respectively). “Other” caregivers, such as siblings, friends, etc., had lower odds of experiencing negative emotional burden than spousal caregivers (OR=.26, CI [.13, .52]; OR=.53, CI [.35, .81], respectively). "Other" caregivers were also 51% less likely to experience social strain than spousal caregivers. Lower odds of experiencing negative emotional burdens were also found with multiple caregivers. The association between adult-child caregivers and social strain was explained by the recipients’ cognitive function. Conclusions: Care-recipient relationship type impacts the QoL in both recipients and their informal caregivers. This association appears to be affected by care recipients' cognitive function level.

